# Improving the Performance of Storage Tank Fault Diagnosis by Removing Unwanted Components and Utilizing Wavelet-Based Features

**DOI:** 10.3390/e21020145

**Published:** 2019-02-04

**Authors:** Viet Tra, Bach-Phi Duong, Jae-Young Kim, Muhammad Sohaib, Jong-Myon Kim

**Affiliations:** School of Electrical, Electronics and Computer Engineering, University of Ulsan, Ulsan 680-749, Korea

**Keywords:** storage tank, fault diagnosis, blind source separation, wavelet-based features, support vector machines

## Abstract

This paper proposes a reliable fault diagnosis model for a spherical storage tank. The proposed method first used a blind source separation (BSS) technique to de-noise the input signals so that the signals acquired from a spherical tank under two types of conditions (i.e., normal and crack conditions) were easily distinguishable. BSS split the signals into different sources that provided information about the noise and useful components of the signals. Therefore, an unimpaired signal could be restored from the useful components. From the de-noised signals, wavelet-based fault features, i.e., the relative energy (REWPN) and entropy (EWPN) of a wavelet packet node, were extracted. Finally, these features were used to train one-against-all multiclass support vector machines (OAA MCSVMs), which classified the instances of normal and faulty states of the tank. The efficiency of the proposed fault diagnosis model was examined by visualizing the de-noised signals obtained from the BSS method and its classification performance. The proposed fault diagnostic model was also compared to existing techniques. Experimental results showed that the proposed method outperformed conventional techniques, yielding average classification accuracies of 97.25% and 98.48% for the two datasets used in this study.

## 1. Introduction

Spherical storage tanks contain fluids, such as liquids and compressed gases, and are widely used in different industries (e.g., the petrochemical, chemical, and aerospace industries) [1,2]. These tanks can be used to store highly hazardous substances that can contaminate the environment easily. However, cracks can develop on the surface of these tanks due to aging, harsh environments, etc. Such cracks can threaten the integrity of the tanks. If a crack on the surface of a tank grows, and the openings of cracks become sufficiently wide, the contained hazardous substance can leak through the cracks and cause air and water pollution [3]. To avoid accidents, it is necessary to manage storage tanks responsibly and periodically inspect them. The traditional procedure for detecting cracks or corrosion in storage tanks is usually carried out on an annual basis via the following steps. First, the storage tank is turned off, emptied, and cleaned. Next, maintenance staff manually identifies any defects, such as cracks or leaks in the storage tank, using either an ultrasonic B-scan or magnetic flux leakage methods. However, the main disadvantages of these processes are the high cost and time-consuming nature.

In recent years, existing techniques that analyze the vibration acceleration signal, motor stator current, and acoustic emission (AE) signal have been widely used in diagnosing bearings [4,5] and commutator motors [6]. Out of these techniques, AE-based techniques are more effective for low speed rotational machines and storage tanks since they are sensitive to the low energy acoustic emissions [7,8]. Acoustic emissions (AEs) are elastic waves generated within a material. The use of acoustic emissions as an alternative tool to detect and locate seeded defects and corrosion in/on the surface of storage tanks, as well as other types of containers working under the pressure of vapor, liquid, or gas like pressure vessels, gas pipelines, and so on, has been adopted by researchers [9,10,11]. The main advantage of this method is that it does not severely interrupt the production process; devices only have to be turned off for a short-term inspection. Moreover, the cost of this method is relatively low compared with traditional methods, which helps businesses save time and money, while minimizing environmental damage. For these reasons, we used the AE signals acquired from the storage tank to determine its condition in this paper. However, due to the complications of the surrounding environment, as well as the limitations of the AE data acquisition device (DAQ), the recorded AE signals were contaminated with noise. As the surrounding environment became more complicated, the noise had a more significant effect on the distribution of the collected signals. Unlike the predictable fault signal, noise sources can produce almost any kind of signal and easily mask the useful signals that characterize the state of a device. These interfering sources can affect signals in both the time and frequency domains; therefore, features extracted from noise-buried signals do not resemble the features of noise-free signals. Moreover, these noise-contaminated features also have a negative impact on the performance of the classifier. To overcome this drawback, we suggested a de-noising method to isolate the original signal from the noise sources.

Traditional de-nosing methods usually rely on frequency characteristics to filter the signal; however, such methods are often prone to obvious disadvantages when used to de-noise non-stationary signals with broadband noise [12]. A state-of-the-art technique that surpasses traditional de-noising methods is the wavelet threshold de-noising method. Nevertheless, this method still does not produce optimal results due to the problem of overlapping frequencies and its difficulty in selecting an appropriate threshold [13]. Similarly, another notable method is empirical mode decomposition (EMD), which has significant noise-reduction ability, but runs into issues related to mode mixing and deficient enveloping [14]. In other words, contemporary de-noising methods are not especially efficient in isolating noise from short-time transient signals. The AE signal collected from sensors is the combination of a useful signal, which is directly related to the health status of a specific object, and diverse exterior and interior interference sources. However, diagnostic systems only utilize characterizing information contained in the useful signal to diagnose an object’s status. In this paper, therefore, we proposed a method to retrieve a noise-free signal that was associated with specific storage tank conditions. To do this, we first separated the observed signal into distinct signal sources using a blind source separation (BSS) technique. These sources were then analyzed to determine whether they were useful or interfering. In this study, we defined useful signal sources as ones that could differentiate between different conditions of the tank. Alternatively, the useless signals (i.e., noise) were sources that appeared every time, regardless of the condition of the tank. These may have been sources coming from the external environment or mechanical noise coming from the interior of the device itself. After removing useless sources, we restored the noise-free signal from the remaining useful components of the signal. Next, features were extracted from the noise-free signals; these were used to identify the different conditions of the tank.

Feature analysis -based fault diagnosis methods have long been used on industrial devices [15,16,17] and have been adopted to detect leakages, cracks, and corrosion in devices working under high pressure [18,19,20]. Typical fault diagnosis methods consist of two basic steps: fault feature calculation and fault classification. Feature calculation (or feature extraction) is an essential step in demonstrating signal statistical characteristics; it is often performed by analyzing the signal in the time domain, frequency domain, or time-frequency domain. In order to reveal underlying properties in the fault-characterizing signal, spectral kurtosis is one of the most cutting-edge methods and is extensively utilized. Its performance in identifying transients was manifested by Dwyer [21] in his early work. Based on the foundation of spectral kurtosis, Antoni and Randall developed its fast realization, the fast kurtogram [22]. The main idea of this work was to find the optimal demodulation band before the use of squared envelope analysis. Following this work, Lei [23,24] proposed the improved and the enhanced kurtograms based on the framework of wavelet packets to diagnose various bearing faults. Barszcz [25] introduced the idea of the protrugram, which calculated kurtosis from the envelope spectrum. From this study, Peter [26] introduced the sparsogram to faster determine the optimal demodulation band. Afterwards, Antoni [27] developed the fast kurtogram to a new method named the infogram, and Wang [28] extended the infograms to novel Bayesian inference. The purpose of the Bayesian inference is to determine optimal wavelet parameters, and thus able to conduct optimal wavelet filtering. Recently, Wang proposed the dynamic Bayesian wavelet transform [29]. The concept of this method was to utilize spectral kurtosis to initialize wavelet parameters and then use dynamic Bayesian inference to find the optimal wavelet transform. In addition to these methods, which try to find an optimal filtering band in order to extract short-time transient properties from the signal, there is another acclaimed approach which extracts features from wavelet coefficients of the wavelet packet transform (WPT). Such features were proved to be sensitive to fault occurrence and robust to different operating conditions. Numerous studies have experimentally demonstrated the effectiveness of these features in diagnosing industrial devices. Zarei and Poshtan, for instance, adopted the WPT to induce a motor’s stator current and calculated sub-frequency band energy to diagnose faults in bearings [30]. Boskoski and Juricic computed Renyi entropy values from coefficients of vibration signals as fault indexes to detect mechanical defects in rotational drives [31]. Li et al. developed a wavelet-based higher-order statistic method, in which Kurtosis values calculated from wavelet coefficients of both the WPT and discrete wavelet transform (DWT) were utilized to detect and classify bearing defects [32]. In this study, we utilized two normalized wavelet packets quantifiers: the relative energy in a wavelet packet node (REWPN) and the entropy in a wavelet packet node (EWPN) as input fault features. REWPN measured the normalized energy of the wavelet packets node, and EWPN defined the uncertainty of the normalized coefficients of the wavelet packets node. Unlike other methods that directly use the amplitude of coefficients, these new quantifiers were derived from probability distributions and were more robust in diagnostic applications [33].

Regarding fault classification, several methods have been introduced and achieved remarkable results, such as a neural network-based method [34,35], fuzzy logic [36,37], and other techniques [38,39]. However, their uses are limited in real-world applications. For instance, network-based classifiers require a sufficient training dataset to generalize and avoid overfitting to deal with complicated and nonlinear problems. On the other hand, although fuzzy logic-based techniques can provide diagnostic outcomes promptly without establishing an appropriate model, their learning abilities are restricted. To address these limitations, we employed one-against-all multiclass support vector machines (OAA MCSVMs), which offered greater classification accuracy with a limited training dataset compared to other classifiers (e.g., artificial neural networks) [40,41,42].

The remainder of this paper is organized as follows. Section 2 demonstrates the experimental test bed of the storage tank and the AE data acquisition system used in this study. Section 3 illustrates the elementary steps of the proposed method for diagnosing abnormal tank conditions. Section 4 exhaustively details the experiments carried out to validate the proposed method. Finally, our conclusions are provided in Section 5.

## 2. The Experimental Test Bed of the Storage Tank and Acoustic Emission Data Acquisition

**Figure 1:**
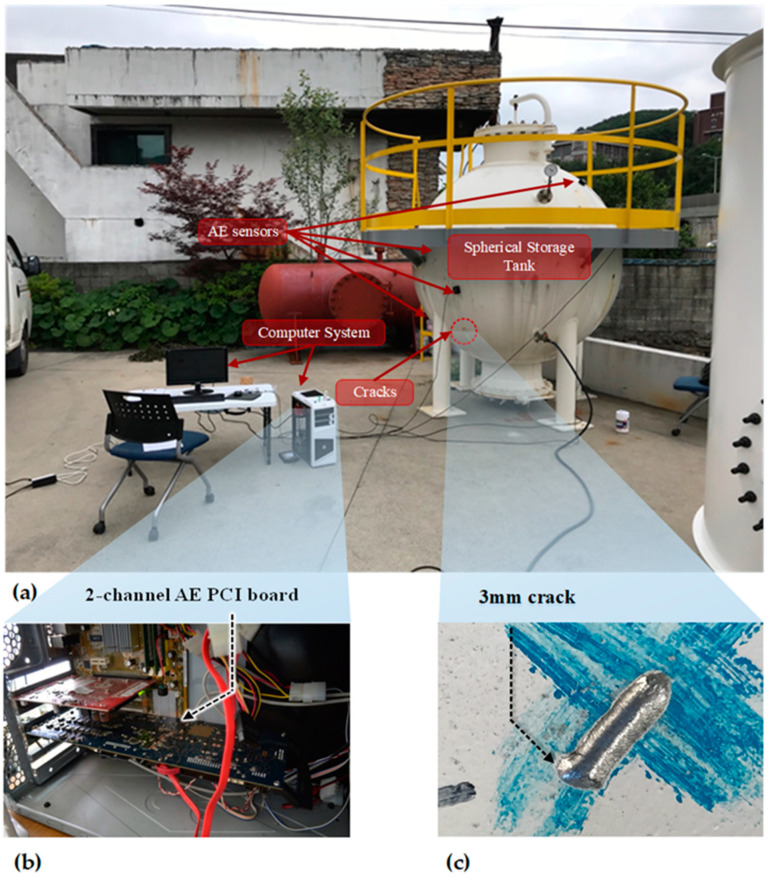
(**a**) The storage tank test bed, (**b**) the data acquisition system using the PCI-2 device, and (**c**) the shape of the crack introduced on the test bed.

**Figure 2:**
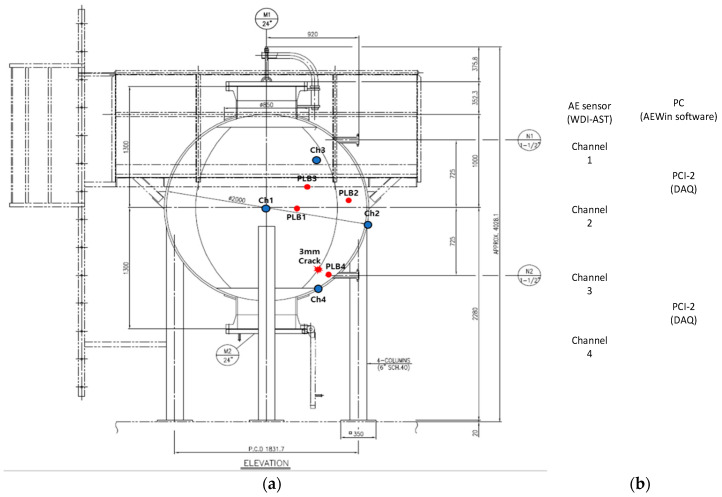
(**a**) Pictorial interpretation of the experimental setup. (**b**) The data acquisition system where PC is a personal computer, PCI-2 represents two channels peripheral component interconnect, and WDI-AST is a wideband differential acoustic emission (AE) sensor.

Experimental data were collected from a test bed consisting of a spherical storage tank made of steel, which contained gasoline, as shown in Figure 1a. In addition, for more details, a schematic diagram is presented in Figure 2a. The tank had a volume of approximately 4.2 m^3^ and a diameter of 2 m. It was loaded under a pressure of 5 bar. In this experiment, we first record AE signals when there was no crack on the wall of the tank; this was done by utilizing four general-purpose wideband frequency AE sensors mounted on the surface of the tank at different locations, which were precisely indicated in the schematic diagram. After completing data acquisition for the crack-free tank condition, we artificially made a 3-mm crack on the surface of the tank bottom to simulate defective conditions in the tank. The shape of the crack is presented in Figure 1c and the distances between the sensors ($x_{1}$, $x_{2}$, $x_{3}$, $x_{4}$) and the crack were 825 mm, 750 mm, 1040 mm, and 430 mm, respectively. Repeating the above data acquisition procedure, we acquired data for the defective storage tank.

To validate the performance of the developed diagnosis model for the storage tank, we sampled the acquired AE signals at 1 MHz to capture very detailed information related to the storage tank condition. This is done with a peripheral component interconnect bus (PCI-2)-based system, as shown in Figure 1b. The connection diagram between sensors and the digitized system is described in Figure 2b, and the technical specifications of AE sensors and the PCI-2-based system are given in Table 1.

## 3. The Proposed Method for Diagnosing Abnormal Tank Conditions

**Figure 3:**
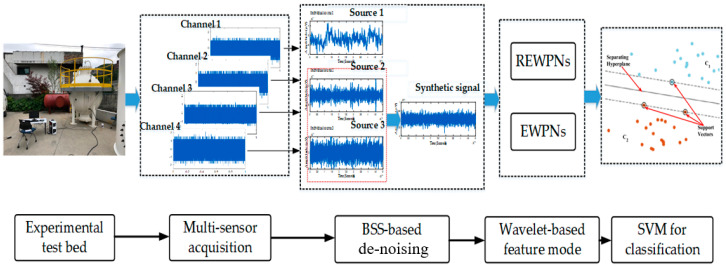
Flowchart of the proposed method for the diagnosis of a storage tank.

The proposed method for detecting fault indications in a storage tank is illustrated in Figure 3, which consists of the following fundamental steps. First, noise-contaminated signals collected via observations were used as the input of a BSS-based denoising mode, where such original signals were split into individual signal sources. These individual sources were then analyzed to determine whether they were useful sources or interference sources. After removing the assumed noise sources, we reconstructed the noise-free signal from the remaining valuable components of the de-noised signal. In the proposed method, wavelet-based fault features that reflect symptoms of different conditions in the storage tank were then extracted from the reconstructed signal. The support vector machine (SVM) trained with such extracted wavelet-based features was then used to classify the “normal” and “crack” conditions of the tank.

### 3.1. Application of Blind Source Separation to Noise-Contaminated Acoustic Emission Signals

It is almost impossible to determine the number of individual sources that are mixed in a measured signal before signal analysis. Therefore, the specific signal separation model utilized in a particular case depends on the correlation between the number of observed signals (sensors) and the actual source (fault and noise) signals. These models can be categorized as determined BSS, over-determined BSS (OBSS), and under-determined BSS (UBSS). This categorization depends on whether the number of observations is equal to, greater than, or less than the number of signal sources, respectively. In practice, fault analysis-related problems usually fall into the case of under-determined BSS because the number of employed sensors is limited, and the noise sources are complex. Among popular approaches, sparse component analysis (SCA), which relies on the sparsity of sources in the time domain or other transformed domains, has been widely utilized to resolve under-determined BSS [43,44]. Several methods rely on the sparsity in the time–frequency domain to separate independent sources [45,46,47]. For instance, in Reference [45], value decomposition was performed on wavelet coefficients to determine frequencies and damping ratios, which makes this method faster than other edge detection approaches. In Reference [46], the principal component analysis was carried out on wavelet coefficients to create the partial mixing matrix of UBSS. In this study, we used the algorithm proposed by Qin [48], which uses a hybrid method combining References [49] and [50], to enhance the efficiency by estimating the mixing matrix and separating individual sources.

#### 3.1.1. Algorithm Description

**Figure 4:**
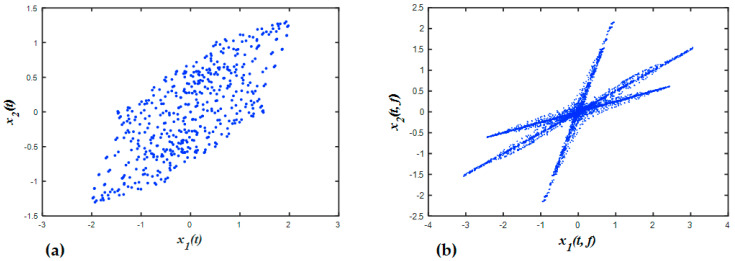
Scatter space of two observations with three sources in (**a**) the time domain and (**b**) the time-frequency domain by STFT.

The most notable features of SCA are the cluster lines in the scattered space, which can be utilized to estimate the mixing matrix. Therefore, it is vital to examine these cluster-based features. Given a numerical example, three harmonic sources $s_{1}(t)=\sin(2\pi *0.8*t)$, $s_{2}(t)=\sin(2\pi *2.5*t)$, and $s_{3}(t)=\sin(2\pi *4.3*t)$ are linearly unified into two observations: $x_{1}=s_{1}+0.3*s_{2}+0.8*s_{3}$ and $x_{2}=0.5*s_{1}+0.7*s_{2}+0.2*s_{3}$. Figure 4a shows the three time–domain observations in a scatter plot. It could be seen that no useful information about the three sources was obtained from this scatter. All the points in the plot were randomly scattered and did not form any distinct cluster that could be used to identify a specific source. Alternatively, when we converted the original signal into the time-frequency domain and plot the real values of the two observations, three distinct clusters were formed. These clusters can be seen as three lines in the scatter pot, where their directions correspond to the column vectors of the mixing matrix, as shown in Figure 4b. By estimating these directions, the mixing matrix can be determined effectively. The regular short-time Fourier transform (STFT) is given as:

(1) $$G(t,\omega )=\int_{-\infty }^{+\infty }g(u-t)*s(u)*e^{-i\omega u}du,$$

where g(u−t) is the step size used to move the window for STFT calculation and s(u) is the truncated signal. The modified STFT with an additional phase shift can be presented as:

(2) $$G(t,\omega )=\int_{-\infty }^{+\infty }g(u-t)*s(u)*e^{-i\omega (u-t)}du.$$

According to Parseval’s theorem, the modified STFT can be rewritten as:

(3) $$G(t,\omega )=\frac{1}{2\pi }\int_{-\infty }^{+\infty }\hat{g}(\omega -\xi )*\hat{s}(\xi )*e^{i\xi t}d\xi ,$$

where $\hat{g}(\omega -\xi )$ and $\hat{s}(\xi )$ are the Fourier transforms (FTs) of the window function and the signal, respectively. With the purely harmonic signal s(t), whose frequency is $\omega_{0}$, $s(t)=A*e^{i\omega_{0}t}$ and ${\hat{s}(\xi )|}_{\xi \neq \omega_{0}}=0$, the STFT of the harmonic signal s(t) can be written as:

(4) $$G(t,\omega )=A*\hat{g}(\omega -\omega_{0})*e^{i\omega_{0}t}$$

For two harmonic signals with different frequencies, $s_{1}(t)=A_{1}*e^{i\omega_{1}t}$ and $s_{2}(t)=A_{2}*e^{i\omega_{2}t}$, their STFT can be written as:

(5) $$\begin{array}{l} G_{s1}(t,\omega )=A_{1}*\hat{g}(\omega -\omega_{1})*e^{i\omega_{1}t}\\ G_{s2}(t,\omega )=A_{2}*\hat{g}(\omega -\omega_{2})*e^{i\omega_{2}t} \end{array}$$

Suppose $x_{1}(t)=a_{11}*s_{1}(t)+a_{12}*s_{2}(t)$ and $x_{2}(t)=a_{21}*s_{1}(t)+a_{22}*s_{2}(t)$ are two observations that are linearly mixed from the signal sources. According to the linearity property of STFT, the STFT of these two observations can be written as:

(6) $$\begin{array}{ll} G_{x1}(t,\omega ) & =a_{11}*G_{s1}(t,\omega )+a_{12}*G_{s2}(t,\omega )\\ & =a_{11}*A_{1}*\hat{g}(\omega -\omega_{1})*e^{i\omega_{1}t}+a_{12}*A_{2}*\hat{g}(\omega -\omega_{2})*e^{i\omega_{2}t}.\\ G_{x2}(t,\omega ) & =a_{21}*G_{s1}(t,\omega )+a_{22}*G_{s2}(t,\omega )\\ & =a_{21}*A_{1}*\hat{g}(\omega -\omega_{1})*e^{i\omega_{1}t}+a_{22}*A_{2}*\hat{g}(\omega -\omega_{2})*e^{i\omega_{2}t} \end{array}$$

We assume $|\omega_{1}-\omega_{2}|>\Delta$, where Δ denotes the frequency support of the window. Therefore,

(7) $$\begin{array}{ll} G_{s1}(t,\omega )\neq 0,\;G_{s2}(t,\omega )=0, & \omega \in (\omega_{1}-\Delta /2,\;\omega_{1}+\Delta /2)\\ G_{s1}(t,\omega )=0,\;G_{s2}(t,\omega )\neq 0, & \omega \in (\omega_{2}-\Delta /2,\;\omega_{2}+\Delta /2) \end{array}.$$

This means that there is no overlap between the two sources in the time-frequency domain. For $\omega \in (\omega_{1}-\Delta /2,\omega_{1}+\Delta /2)$, the ratio of the two signal sources in time frequency domain is given as:

(8) $$\frac{G_{x1}(t,\omega )}{G_{x2}(t,\omega )}=\frac{a_{11}*G_{s1}(t,\omega )}{a_{12}*G_{s1}(t,\omega )}=\frac{a_{11}}{a_{12}},$$

and, for $\omega \in (\omega_{2}-\Delta /2,\omega_{2}+\Delta /2)$, the ratio is:

(9) $$\frac{G_{x1}(t,\omega )}{G_{x2}(t,\omega )}=\frac{a_{21}*G_{s1}(t,\omega )}{a_{22}*G_{s1}(t,\omega )}=\frac{a_{21}}{a_{22}}.$$

Equations (8) and (9) indicate that, for the linear mixture, we can plot the STFT of two observations into the scatter, i.e., G(t,ω)x1 versus G(t,ω)x2 in each frequency bin. These lines have the same direction, which is determined by the amplitude of sources mixed in the observations, i.e., the column vector of the mixing matrix. This explains why some cluster lines appear in the scatter plot, which can be employed to obtain the mixing matrix.

#### 3.1.2. Application of BSS to Noisy Acoustic Emission Signals

Classifiers only use characteristics related to the health status of a device to identify the condition of the device. Therefore, the idea in this paper was to separate the composite signals obtained from observations into individual signal sources. These individual sources could then be assessed to identify whether a source represents a useful signal or a noise source. Useful signals are ones that can highlight differences between the different conditions of the tank. In contrast, the useless signals, or noise, appear in all the conditions of the tank. Therefore, noise is eliminated, and only useful signals are retained. The obtained de-noised signals are then subjected to a feature extraction process to create a feature pool that can characterize different conditions of the storage tank.

**Figure 5:**
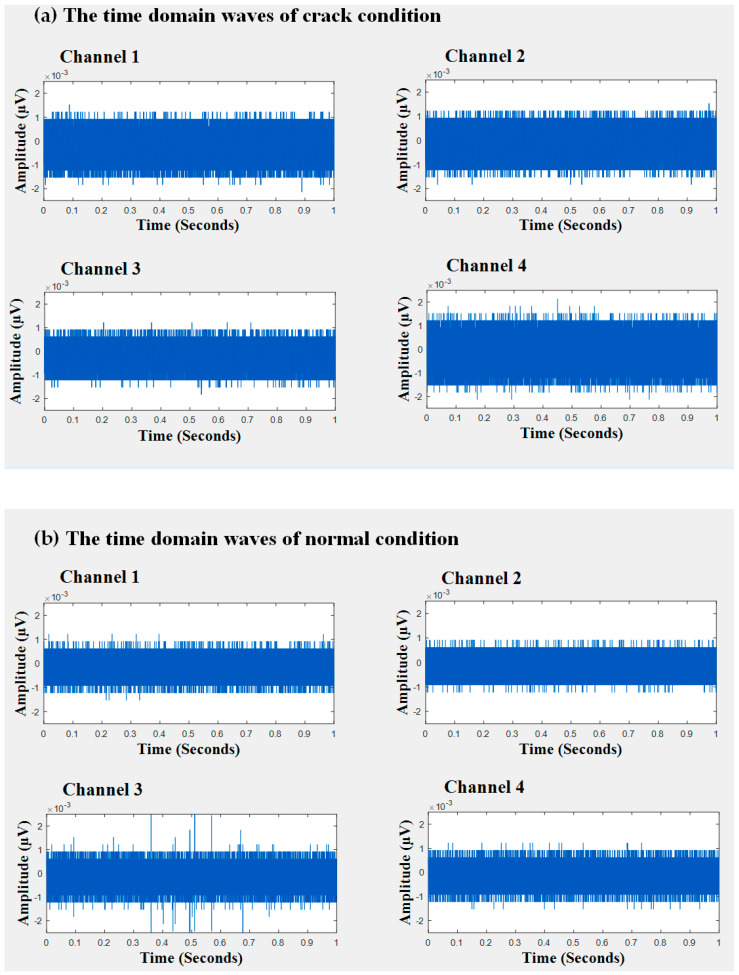
The time domain wave of observations: (**a**) crack condition and (**b**) normal condition.

**Figure 6:**
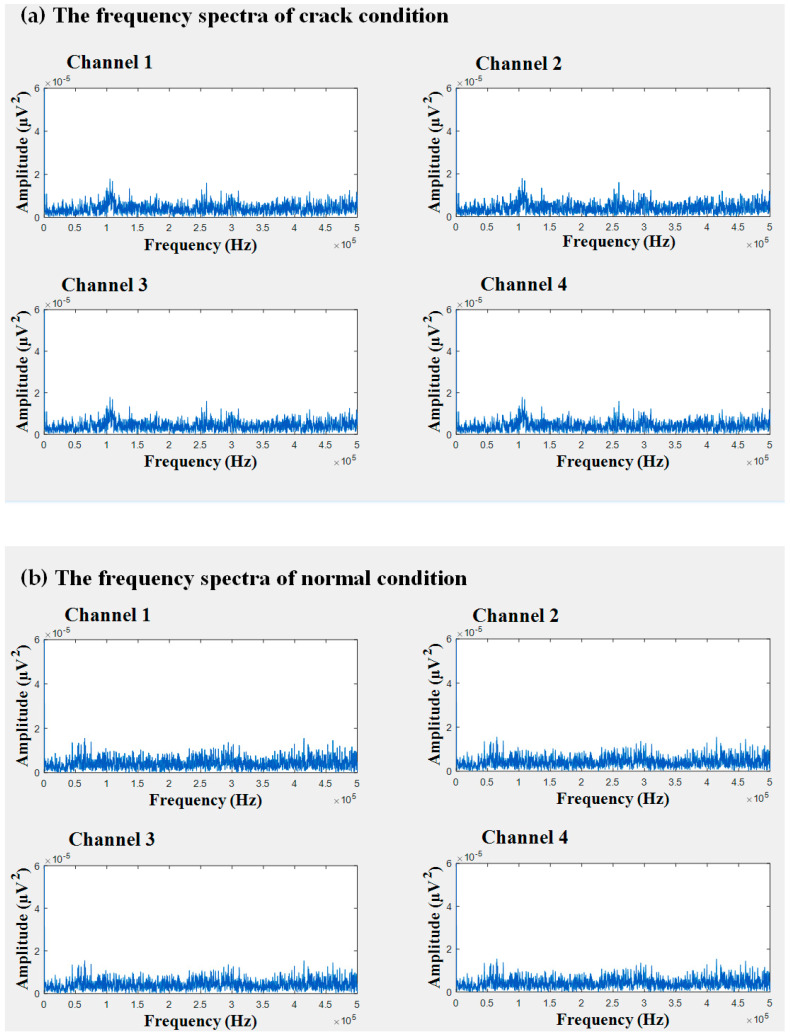
The frequency spectrum of observations: (**a**) crack condition and (**b**) normal condition.

In this study, AE signals were collected from four different sensors mounted on the wall of the storage tank. The time domain and frequency domain illustrations of these observations are presented in Figure 5 and Figure 6, respectively. From the time domain waveforms and frequency spectrograms, it was obvious that procuring useful information is difficult unless and until the signals have been preprocessed. To de-noise the acquired AE signals from the healthy and defective conditions of the tank, we used the BSS algorithm mentioned in Section 3.1.1. Through the BSS algorithm, we were able to decompose signals of faulty conditions into three individual signal sources. Likewise, signals of the normal condition could be split into two individual signal sources through the de-noising algorithm.

**Figure 7:**
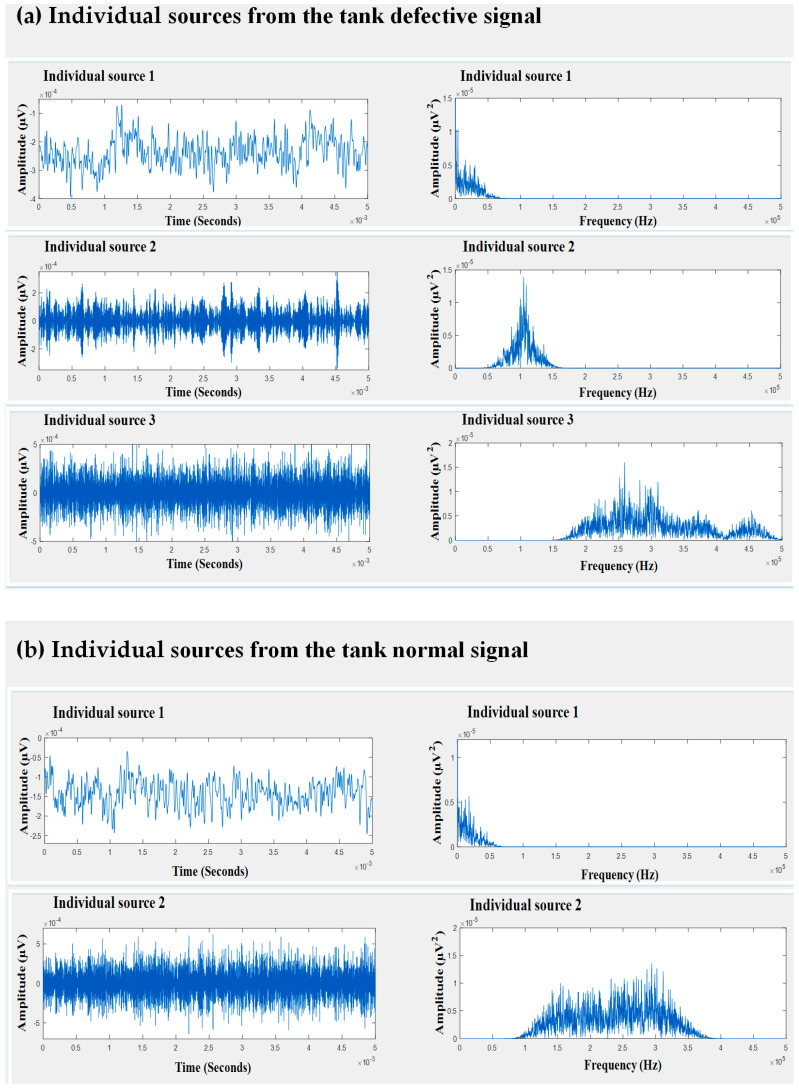
The time domain waves and spectra of recovered sources: (**a**) crack condition and (**b**) normal condition.

**Figure 8:**
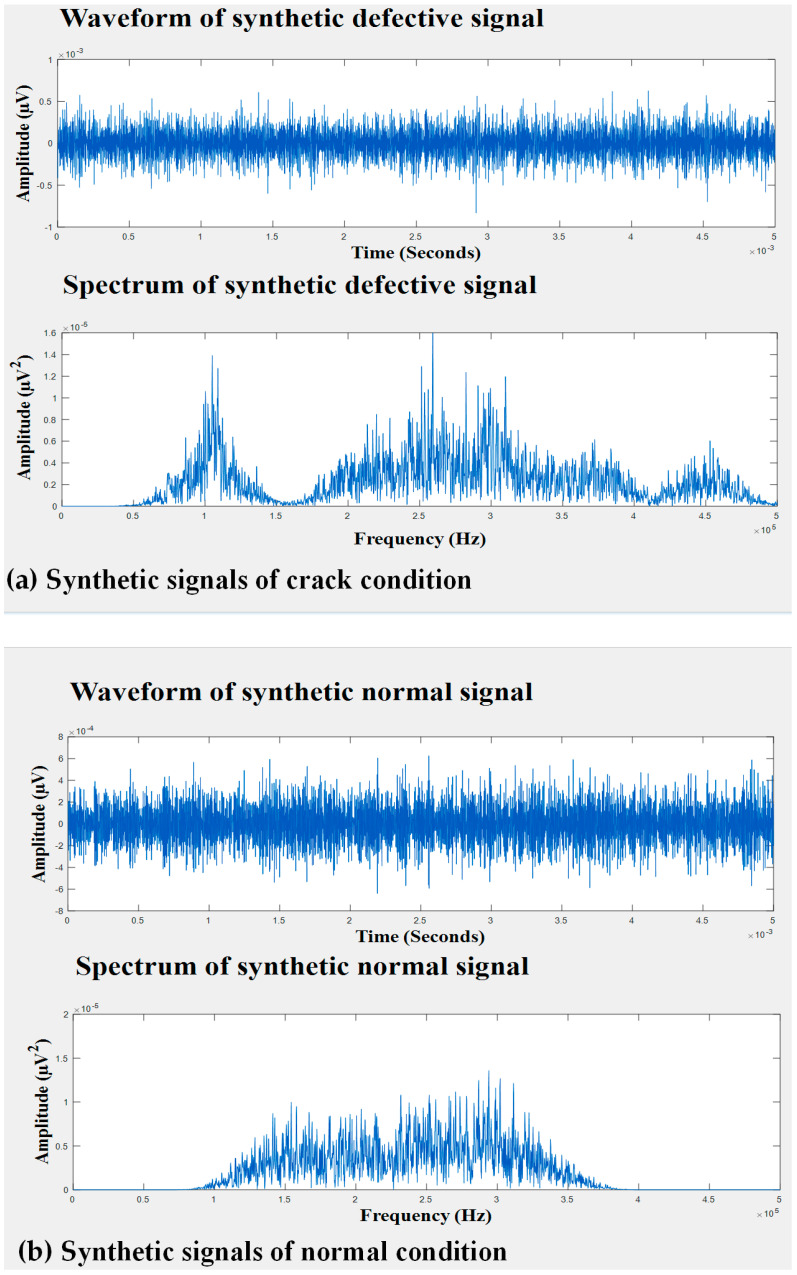
The time domain waves and spectra of synthetic signals: (**a**) crack condition and (**b**) normal condition.

It can be seen in Figure 7 that the individual sources separated from both the faulty signal and normal signal were similar. This suggested that this source was not associated with either of the tank conditions; instead, it may have been caused by external interference. Therefore, it was safe to eliminate this signal source. In contrast, the remaining sources helped to improve the discriminative characteristics between the two tank conditions. Therefore, they were retained to reconstruct noise-free signals that could define the behavior of the different conditions of the tank. Figure 8 shows the waveforms of the recovered signals in the time domain. The recovered signals were also transformed into the frequency domain in order to better examine their characteristics, as presented in Figure 8. Later, wavelet transform features were extracted from the de-noised signals and provided to the classifier.

### 3.2. Feature Calculation

**Figure 9:**
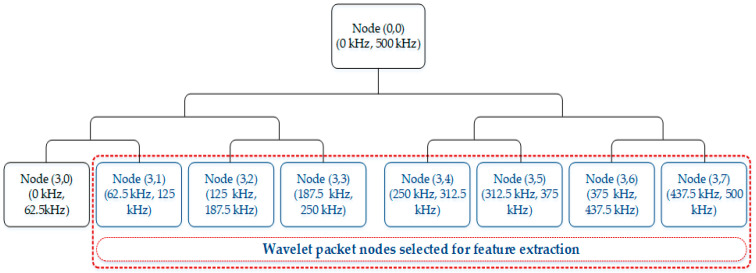
Three-level wavelet packet transform (WPT) on an AE signal.

To collect AE signals for identifying non-stationary tank defects, AE sensors were attached to different locations of the tank wall. Due to the attenuation in the measured AE signals caused by the distance between crack sources and sensors, underlying information related to the storage tank failures mainly lies on the mid-frequency and high-frequency bands. The WPT, with its decomposition ability that can split a signal into low-frequency and high-frequency bands, has been widely utilized as a favorable time-frequency analysis tool for short-time transient AE signal processing. Therefore, in this study, WPT was used to analyze fault features by computing the REWPN and EWPN [51]. These fault features provided promising details regarding fault symptoms in fault diagnosis applications. To calculate these features, we decomposed a one-dimensional AE signal into eight wavelet packet nodes via three-level WPT with the help of Daubechies 20 (or db20). From Figure 8, it is noticeable that the inherited information regarding faults in the AE signals of the tank was mostly located in the mid-frequency and high-frequency bands. Thus, we used wavelet-based fault features (REWPN and EWPN) that were extracted from the last seven mid-frequency and high-frequency wavelet packet nodes, as shown in Figure 9. The REWPN of the nodes was calculated as follows:

(10) $$REWPN(i)=\frac{\sum_{j=1}^{K}w_{i,j}^{2}}{\sum_{n=1}^{N_{tnode}}\sum_{j=1}^{K}w_{n,j}^{2}},$$

where $N_{tnode}$ was the total number of wavelet packet nodes analyzed in this study ($N_{tnode}=7$), and $w_{i,j}$ was the $j_{th}$ wavelet coefficient out of a total of K wavelet coefficients from the $i_{th}$ wavelet packet node. The formula used to calculate EWPN was:

(11) $$EWPN(i)=-\sum_{j=1}^{K}p_{i}(j)\log_{2}p_{i}(j),$$

where $p_{i}(j)=w_{i,j}^{2}/\sum_{j=1}^{K}w_{i,j}^{2}$. A total of 14 wavelet-based features, consisting of seven REWPNs and seven EWPNs, were utilized in this study to diagnose tank defects.

### 3.3. Fault Classification

An SVM is a discriminative classifier officially defined by a hyperplane that separates two classes with the largest margin in the feature space. To find the ideal hyperplane that separates two classes, we needed to solve the optimal minimization problem:

(12) $$\begin{array}{ll} \underset{{w,\xi }_{i}}{\mathrm{argmin}}\left\{\frac{1}{2}w^{T}w+C\sum_{i=1}^{n}\xi_{i}\right\},\\ & ,\\ \begin{array}{ccc} & \mathrm{subject}\;\mathrm{to} & \end{array}y_{i}(w^{T}\phi (x_{i})+b)\geq 1-\xi_{i},\xi \geq 0,\forall i=1,2...,n \end{array}$$

where w was the normal vector to the hyperplane, ϕ was a nonlinear function used to project the initial feature space into the high-dimensional nonlinear feature space, C was a penalty variable used to tune the generalization capability, and $\xi_{i}^{}$’s were the slack variables used to regulate the training error. Using Lagrange multipliers, the optimal minimization problem can be solved as follows:

(13) $$\begin{array}{ll} \underset{\alpha_{i}}{\mathrm{argmax}}\left\{\sum_{i=1}^{n}\alpha_{i}-\frac{1}{2}\sum_{i=1}^{n}\sum_{j=1}^{n}\alpha_{i}\alpha_{j}y_{i}y_{j}K(x_{i},x_{j})\right\},\\ & ,\\ \begin{array}{ccc} & \mathrm{subject}\;\mathrm{to} & \end{array}\;\sum_{i=1}^{n}\alpha_{i}y_{i}=0,0\leq \alpha_{i}\leq C\;\forall i=1,2,...,n \end{array}$$

where $\alpha_{i}’$s are Lagrange multipliers and $x_{i}$ and $x_{j}$ present samples in the training dataset. In this study, we used the Gaussian radial basis function (RBF) kernel function for the SVM due to its satisfactory performance. The RBF kernel is computed as follows:

(14) $$k(x_{i},x_{j})=\exp(-\gamma ||x_{i}-x_{j}||),$$

where $\gamma =1/2\sigma^{2}$ and σ are adjustable parameters that can be cautiously tuned. If σ is small, the exponential is linear, and the higher dimensional mapping loses its nonlinear power. In contrast, if σ is large, the decision boundary is very sensitive to noise during training, which leads to a lack of regularization.

In this study, a one-against-all scheme was used to design multiclass support vector machines (OAA MCSVMs). This scheme was useful due to its simplicity and ability to solve multiclass classification problems. Each SVM structure in the OAA scheme separated one class from the others, and the SVM structure that offered the highest output value was selected to make the final decision. In addition, during the OAA MCSVMs training process, each SVM structure was individually evaluated to gain the highest classification accuracy for its own class. The SVM structures were analyzed together to make a final decision.

## 4. Experimental Result and Discussion

### 4.1. Configuration of Training and Testing Data

To validate the effectiveness of the proposed approach, we used two datasets corresponding to crack sizes of 3 mm and 6 mm on the storage tank wall (Table 2). Both datasets contained AE signals for a storage tank with a crack located on its wall and a defect-free tank, i.e., a total of two different tank conditions. For each dataset, the *k*-fold cross validation (*k*-cv) approach was employed to calculate the generalized classification accuracy. In *k*-cv, the dataset utilized for evaluating the diagnostic capability of the proposed method was first divided into *k* mutual folds, which were denoted as $D_{1},D_{2},...,D_{k}$. At the *j*-th iteration in *k*-cv, fold $D_{j}$ was reserved as the testing set, while the remaining folds were utilized to train OAA MCSVMs. The classification accuracy was successively estimated *k* times by training and testing OAA MCSVMs, and the final classification accuracy was the mean value of the accuracies obtained in each fold.

A *k*-cv scheme was used to evaluate the developed OAA MCSVMs, and the value of *k* was set to 3. Therefore, an initial sample set containing a total of 1080 samples from the two conditions of the tank (i.e., 540 samples for each condition) was split into three subsets (each fold included 360 samples for each condition). To determine the classification performance of the methodology, each of the three subsets was used as the testing set and the other two were used as the training set. The individual classification accuracy was determined by testing OAA MCSVMs in the given fold, and the final classification accuracy was the average value of the three individual accuracies calculated in each fold.

### 4.2. Efficacy of Removing Useless Elements

**Figure 10:**
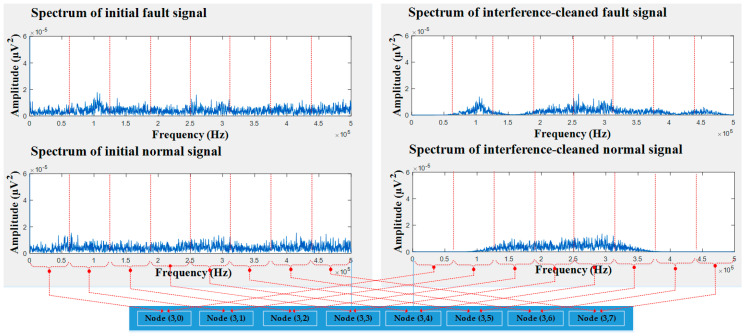
The differences in input signals after being processed.

To demonstrate the effectiveness of the proposed scheme, we visualized the input signal in the frequency domain before and after removing noise, as shown in Figure 10. It was noticeable that the two tank conditions, i.e., a normal state and faulty state, were easily distinguishable after eliminating noise. To be specific, wavelet packet nodes from two types of signals showed a clear difference from each other. When a classifier was trained with such discriminating features, it yielded higher classification accuracy. Alternatively, original signals without preprocessing were contaminated with noise; therefore, these signals were undistinguishable.

In addition to visualization results, we also provided qualitative evaluation of the diagnosis system by classifying the performance when original signals without preprocessing were used with the model. In this study, we used the *k*-cv scheme to partition the dataset into *k* subsets, where *k* = 3 in this case, the individual classification accuracy was measured using two subsets for training and the remaining subset for testing OAA MCSVMs. The individual classification accuracy was calculated as follows:

(15) $$C_{accuracy}=\frac{\sum_{L}N_{TP}}{N_{samples}}\times 100(\%),$$

where L was the number of classes, i.e., L=2; $N_{TP}$ was the number of true positive (TP) instances, which was the total number of samples in class i of the testing set that were exactly classified as class i; and $N_{samples}$ was the total number of samples in the testing set, i.e., $N_{samples}=360$. The performance of the proposed tank fault diagnosis model was evaluated using the average classification accuracy (ACA), which was the mean value of the three individual accuracies calculated using the *k*-cv scheme. Table 3 presents the average classification accuracies of the fault diagnosis models. According to Table 3, it could be seen that the proposed fault diagnosis method using the signal de-noising scheme yielded better average classification accuracies than the one that did not use signal pre-processing steps. The noise-contamination was the main reason that the classifier faced difficulties in analyzing the fault information inherited in the original signals, which resulted in classification accuracy degradation.

### 4.3. Efficacy of the Wavelet-Based Features

In this study, the feature pool values created from wavelet-based attributes were used as inputs for the standard OAA MCSVMs. The reason behind this choice is that it can be used to catch underlying properties in short-time transient signals by splitting a signal into low-frequency and high-frequency bands. This decision was also made based on the apparent difference between frequency bands or wavelet packet nodes of two tank signal types, i.e., a normal tank signal and a defective tank signal. To validate our scheme, it was necessary to compare our model’s classification performance with a model that uses another fault feature set. The rival in this experiment was a fault feature vector with different statistical measures of the time and frequency domain [15]. Table 4 presents the diagnostic performance of the proposed model and the fault diagnosis model with a different feature pool (i.e., different time and frequency features). The diagnostic performance of all the methods was measured in terms of the ACA mentioned in Section 4.2. From the results given in Table 4, it could be concluded that the proposed model yielded greater diagnostic performance than the other method. This means that the feature set used in this study provided better discrimination between the signal types compared to the other feature set using different time and frequency features. The ACA of the proposed method was 98.48%, which showed a 4.76% improvement compared to the method which used the different types of feature vectors (i.e., 93.72%).

**Figure 11:**
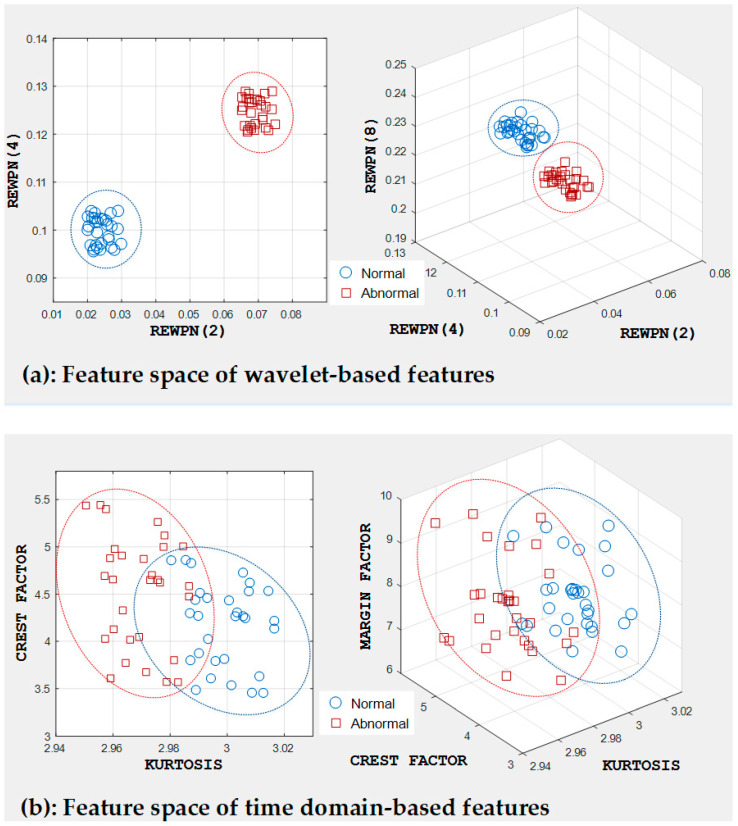
Feature space of (**a**) wavelet-based features and (**b**) time domain-based features.

The effectiveness of the suggested input features is also evident in Figure 11, which shows the distribution of various features in the three-dimensional feature space. As shown in Figure 11a, the WPT-based features form distinct clusters helped the classifier to easily draw a hyperplane between the samples of different classes in order to classify them. In contrast, when we used features in the time and frequency domain, some samples that belong to different categories overlapped, as shown in Figure 11b, which deteriorated the diagnostic performance of the classifier.

Moreover, besides selecting the appropriate fault feature set, choosing good vales for the penalty and kernel parameters (C,σ) in OAA MCSVMs, as mentioned in Section 3.3, was also critical. We employed a grid search technique to decide the optimal combinations of these parameters in the SVM structure in terms of the classification performance. The grid search algorithm trained OAA MCSVMs with a pair (C,σ) in the cross product of the following two sets and evaluated their performances: $C\in \{2^{-5},2^{-3},2^{-1},2,2^{3},2^{5},2^{7},2^{9},2^{11},2^{13},2^{15}\}$ and $\sigma \in \{2^{-2},2^{-1},2,2^{2},2^{3},2^{4},2^{5},2^{6},2^{7}\}$. Finally, the pair (C,σ), which helps OAA MCSVMs deliver the highest classification performance, was considered as the best combination of these parameters.

## 5. Conclusions

This paper proposed a new approach to effectively discriminate between two different types of signals taken from a spherical tank based on a BSS technique. This discrimination between the signals, therefore, improved the reliability of the fault diagnosis system developed for a storage tank. Moreover, this paper presented WPT-based fault features that had an ability to explore salient information in nonstationary AE signals. These features were then used to train OAA MCSVMs. Experimental results indicated that the proposed tank fault diagnosis method achieved a higher classification accuracy of 98.48% compared to other two-fault diagnosis models. In a fault diagnosis model without a signal preprocessing step and a model using a different feature set (i.e., features in the time and frequency domain), the yields were 90.38% and 93.72%, respectively.

In the future, we will explore several state-of-the-art techniques to compare and validate the effectiveness of the proposed method since some notable methods described in the introduction section can exploit hidden characteristics of such nonstationary signals.

## Figures and Tables

**Table 1:** Detailed specifications of the data acquisition system.

**AE sensor (PAC WSα)**	▪ Peak sensitivity (V/μ bar): −62 dB ▪ Operating frequency range: 100–900 kHz ▪ Resonant frequency: 650 kHz ▪ Directionality: ±1.5 dB
**Two-channel AE PCI board**	▪ 18-bit 40 MHz A/D conversion ▪ 10 M samples/s rate as one channel is used (5 M samples/s as two channels are used simultaneously)

**Table 2:** Details of the two datasets utilized to evaluate the proposed methodology.

*f_s_* = 1 MHz		No. Samples ^1^	Crack Size		
			Length (mm)	Width (mm)	Depth (mm)
Dataset 1	Training set	1080	3	0.5	0.4
	Testing set			0.5	0.4
Dataset 2	Training set	1080	6	0.7	0.5
	**Testing set**			**0.7**	**0.5**

^1^ For each tank condition, i.e., a defect-free tank and defective tank, 540 feature vectors are used.

**Table 3:** The average classification accuracies (ACAs) of the proposed method and the fault diagnosis model without using signal pre-processing.

Datasets	Methodologies	Average Accuracy (%)
Dataset 1	[*]	90.15
	Proposed	97.25
Dataset 2	[*]	90.38
	Proposed	98.47

[*] is the classification model that uses a noise-contaminated signal as the input.

**Table 4:** The average classification accuracies (ACAs) the proposed method and the counterpart.

Datasets	Methodologies	Accuracy (%)
Dataset 1	[**]	92.35
	Proposed	97.25
Dataset 2	[**]	93.72
	Proposed	98.48

[**] is the classification model that uses time and frequency domain-based fault features.
